# Impact of Advance Care Planning Support on Patients Treated in the Intensive Care Unit After High-Risk Surgery

**DOI:** 10.7759/cureus.54175

**Published:** 2024-02-14

**Authors:** Kanako Yamamoto

**Affiliations:** 1 Critical Care Nursing, St. Luke's International University, Tokyo, JPN

**Keywords:** surgery, decision aid, decision-making, shared decision making, advance care planning, intensive care unit

## Abstract

Introduction: Efforts to enhance support for advance care planning (ACP) in patients requiring emergency and intensive care are currently being explored. In addition, few studies have reported the effects and impact of support for these patients and their families. The researcher developed a patient decision aid to initiate support for ACP for patients who plan to enter the intensive care unit after surgery and their families. This study aimed to provide ACP support to patients before high-risk surgery and to determine its impact.

Methods: The study design was qualitative. The participants included 10 patients who were scheduled to be admitted to the intensive care unit after high-risk surgery at an acute-care hospital in Japan, and the patients’ families. The researcher used decision aids to implement ACP support before the patients were admitted. Participants were interviewed in a semi-structured manner regarding their experiences and the impact of receiving ACP support after discharge. Interviews were recorded using an integrated circuit recorder, followed by verbatim transcripts. The analysis was performed in a qualitative descriptive manner.

Results: ACP support prior to treatment initiation led patients to think about life-sustaining treatments and consider ideal living. By understanding the risks of treatment, patients can calmly assume complications and discuss their mortality and life after surgery. Patients perceived receiving ACP support as a valuable benefit prior to undergoing treatment in the intensive care unit. After discharge, they wanted to promote shared decision-making among their physicians. On the other hand, family members were more anxious about ACP topics than patients. In addition, the patients and their families felt that it was difficult to discuss their thoughts and wishes regarding ACP before surgery.

Conclusion: It is suggested that pretreatment ACP support could serve as an introductory phase for patients anticipating the need for intensive care, allowing them to contemplate their preferences regarding life-sustaining treatment. However, it is difficult for patients and their family members to openly discuss their thoughts on life-sustaining treatment, even if they are aware of the risk of a sudden crisis. Therefore, when patients and their families discuss ACP, the inclusion of healthcare coaching and counseling may be more effective. These measures of ACP support could add to increased family discussions, concordance, and shared decision-making with physicians.

## Introduction

Owing to advancements in medical care, older and high-risk patients with medical histories have increased opportunities for surgery [1,2]. As a result, they have more opportunities to receive emergency and intensive care [3,4]. Patients who undergo high-risk surgery or intensive care, along with their families, are in a stressful state [5]. This is due to the increased emotional burden associated with the risk of postoperative complications and intraoperative mortality [6,7]. Patients may undergo unexpected and rapid critical changes and transition to end-of-life care [4,8]. In such cases, medical professionals often feel that it is difficult to think about better treatments for patients [9].

Studies clarifying the effect of advance care planning (ACP) support on patients receiving emergency and intensive care have been reported [10,11]. ACP is a process wherein a patient envisions end-of-life care and discusses with a physician or family member the treatment preferences they desire, starting from a healthy age [12]. The goal of ACP is to improve the quality of end-of-life care [12,13]. Thus, there are few opportunities for ACP support in surgery and intensive care, which are considered aggressive treatment options. Surgeons and intensivists worry that providing ACP support before treatments such as surgery may increase anxiety or treatment refusal for patients and/or their families [9,10]. Therefore, ACP support is sometimes considered inappropriate for patients undergoing aggressive treatments [10].

Few studies have evaluated the implications of discussing ACP before treatment for patients and their families who are not foreseeing end-of-life situations [14]. It is becoming recognized that ACP support for patients undergoing high-risk surgery is a necessary and important topic for providing patient-centered care [15]. However, this is a sensitive topic, and the direction of support is still being explored [16]. Therefore, this study aimed to understand how providing ACP support impacts high-risk surgical patients requiring critical care and their families. Our research questions are as follows: What are the common themes identified for non-terminal patients after receiving ACP support, and how can the effects and disadvantages of ACP support prior to surgery inform the development of a specific measure for support?

This article was previously presented as a meeting abstract at the Best of SCCM Congress 2022 Taipei on October 15-16, 2022.

## Materials and methods

 Study design and participants

This study included patients scheduled to be admitted to the intensive care unit (ICU) after surgery, as well as their family members. The facility was a Japanese acute care hospital. There were three eligibility criteria: 1) patients must be at least 20 years of age and able to communicate in Japanese, 2) permission from the physician must be obtained, and 3) each patient has decided to undergo surgery. Two additional exclusion criteria were applied: 1) a history of dementia or current cognitive decline and 2) emergency surgery. Family members designated by the patients were enrolled, and their consent was obtained.

The study was conducted from September 2021 to February 2022. This study was part of a study on ACP implementation in patients treated in the ICU and their families [11]. This study aimed to understand the experiences of patients receiving ACP support prior to surgery and their families. The need for ACP support among medical professionals for patients undergoing high-risk surgeries remains controversial. This is partly because of the lack of information on the effects of ACP on patients who undergo high-risk surgery and their families. Therefore, a qualitative descriptive research method was employed [17].

Study process

**Figure 1:**
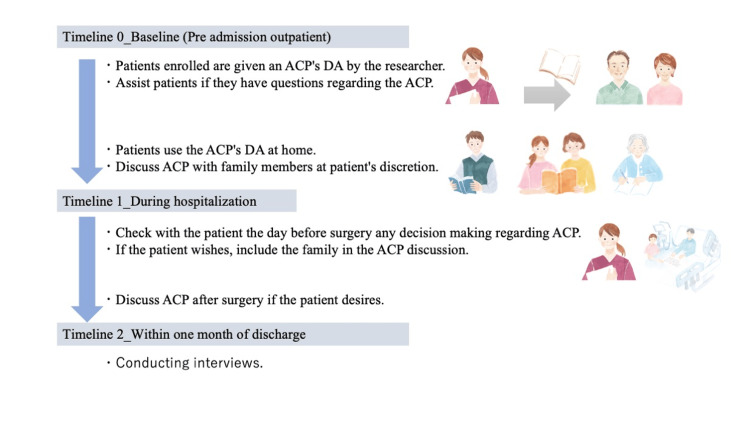
Flowchart of the study ACP, advance care planning; DA, decision aid.

The process of this study is illustrated in Figure 1. The purpose of the study was explained to the patients who decided to undergo surgery. ACP support was initiated when the patients provided consent. First, patients were provided with decision aids (DAs) for ACP at outpatient visits. In addition, patients were instructed to use DAs before admission. Next, the researchers met with the patients when they were admitted to the hospital to share their thoughts or wishes regarding ACP and provide the necessary counseling for decision-making. During hospitalization, patients contacted the researchers if they requested consultation regarding ACP. Finally, they were interviewed about their experiences of thinking about and making decisions regarding ACP after discharge.

DA used for Intervention

This study utilized a researcher-developed ACP DA [11]. It was developed according to a systematic model development process and satisfied international standards [10]. This DA consists of two chapters: Chapter 1) The patients consider the medical care they want to receive and discuss it with their families. Chapter 2) The patients consider the medical care they would like to receive in a sudden critical situation, including “do not attempt resuscitation” (DNAR). This DA was designed to allow patients to step through the ACP process and consider it at their own pace. The DA is a paper-based booklet that the patient could write in while reading.

Data collection

Patients were considered candidates if they decided to undergo surgery. After the patients visited their physicians, the researcher explained the study in writing and verbally. The patients were given time to participate in the study. The researcher was contacted individually if the patients agreed to participate. The patient’s family members were also provided with written and oral explanations and were contacted at a later date if research assistance was available. Patient interventions started before admission, and the researcher was contacted on and during admission. Interviews were conducted within one month of discharge. After patients’ outpatient visits, a researcher contacted them and interviewed them in a private room in the hospital.

The researcher conducted semi-structured interviews for approximately 30 min using an interview guide. Interviews were generally conducted one-on-one; however, if the patients requested it, the researcher interviewed the patient and their family members simultaneously. Interviews were conducted in private rooms, and conversations were recorded using a digital voice recorder. The patients were asked, “How has the experience of considering ACP before surgery affected you?” Families were asked, “What impact did the patient’s consideration of ACP before surgery have on you?” Medical records were also reviewed with participant consent. Data collected included sex, age, disease, surgical name, length of hospitalization, and ICU stay.

All interviews were conducted by the authors. The interviews may have caused the participants to recall negative or distressing memories during their hospital stay, which may have caused emotional strain. Therefore, the interviews were conducted with great care, and the participants were fully informed in advance that they were not pressured to talk and that they could stop at any time if they felt mentally burdened.

Data analysis

Data from the interviews were recorded verbatim and analyzed qualitatively. The authors conducted all the interviews and were involved in the analysis to eliminate bias. Patient data were managed using ID numbers, and verbatim transcripts were created by a private company. The verbatim transcripts were read, and the text was interpreted in terms of how the participants reviewed the ACP and what the experience meant. Codes were then generated using the text data and compared accordingly. Codes with the same meanings were collected, raised in the abstract, and grouped into subcategories. Similar subcategories were grouped and abstracted into categories. Qualitative researchers supervised the analyses. In addition, the results of the analysis were reviewed by the researchers and supervisors without knowing the descriptions of the participants, and the analysis was checked for bias. The validity and reliability of the results were discussed between researchers and supervisors until a unified interpretation was obtained. This manuscript adhered to the standards for reporting qualitative research guidelines for reporting qualitative studies [18].

Ethical consideration

This study was approved by the Ethical Board of St. Luke’s International University (approval number: 21A007). The participants provided consent for the purpose of the study, methods of investigation, participation in and exclusion from the study, protection of personal information and data management, access to medical records, publication of results, and signing of documents. The acquired data were managed separately, and ID numbers and correspondence tables were created so that the individuals could not be identified.

## Results

Twenty participants, comprising 10 patients and 10 family members, took part in this study. The average interview duration was 41.6 min. Table 1 provides a summary of the participants.

**Table 1:** Demographic details of participants SD, standard deviation; ICU, intensive care unit

Variable	Patients	Families
	n=10	n=10
Sex		
Female	6	5
Male	4	5
Age, mean±SD	56.9±10.4	50.1±14.1
Employment		
Full-time	6	8
Part-time	1	1
Unemployed	3	1
Disease		
Cardiovascular	7	
Gastroenterological	3	
Patients with cancer	6	
Mean ICU length of stay, mean±SD	1.8±1.3	
Mean hospital length of stay, mean±SD	8.2±2.5	
Relationship with family		
Partner	7	
Children	3	

Patients’ experiences

Five patients spoke to three parties, including family members, at their request. After the analysis, eight categories and 40 subcategories were extracted (Table 2). Patients received ACP support before surgery to reflect on their wishes for life-sustaining treatment and indicate their need for active participation in decision-making. Additionally, rather than making decisions in discussions with someone else, patients made their own choices first and then confirmed them by telling the family members that these are the best choices.

**Table 2:** Patients' experiences considering advance care planning before surgery ACP, advance care planning; DA, decision aid.

Category	Subcategory
Active participation in decision-making and expression of discretion.	I want to make my own decisions.
	Reconsider who one shares my thoughts with.
	I can't start discussing my thoughts with anyone until I decide.
	Awareness of the possibility of thoughts conflict with family or physician.
	Realize that it is important to communicate my thoughts on my own.
Having the opportunity to share my thoughts with family.	Recognize the meaning of expressing my thoughts to my family.
	I could tell my family one-sidedly, but it's hard to discuss.
	Aware of the family's values.
	It's hard to hear my family's true thoughts.
Thinking about the wishes and goal of life-sustaining treatment for myself.	ACP's decision go wobbly with every change in treatment or disease.
	I want to reconsider the intention of ACP according to the treatment progress and age.
	Seek a premise for making a final decision.
	Marshal my values.
	Be prepared in advance to clarify thoughts and reasons.
	Be able to materialize the wishes of life-sustaining treatment for myself.
	It's hard to come to a final conclusion when I sort out my own values.
Understands the worst risks, prepares for ACP, and has surgery.	I want my doctor to approve my decision.
	I think I made the best choice I can make now.
To start planning for life after treatment.	Understand the need to think about and prepare for ACP.
	It gives me a chance to think about what to look forward to after treatment.
	Think about who to talk to, when and to what extent using DA.
	Revisit and think about it repeatedly after surgery.
	Estimate the course of treatment and plan who to share thoughts with and to what extent.
	I can share with my family the need to understand the ACP process.
	I am worried about whether they will empathize with me after I tell them.
	Thinking about ACP once will prepare me for future treatment.
Understand the meaning of considering ACP before surgery.	ACP thought about before surgery will help me make my own treatment decisions in the future.
	Before surgery is a good time to think about how I want to live my life.
	Thinking about ACP before surgery makes me feel sad.
	Worried about whether the surrogate decision-maker can make decisions on my behalf.
	It is an opportunity to talk with family members.
	It gives me a chance to tell my family my true thoughts about the disease and treatment.
I'd like to promote shared decision making with a physician.	Plain-speaking discussions about life-sustaining treatment are less likely to lead to misunderstandings.
	Understand what it means to find a doctor I can trust.
	It's also important to allow the topic of life-sustaining treatment to come up.
	Hopefully ACP will be included as part of perioperative care.
	The topic of ACP before surgery is not a good time to explore the doctor's reaction and say what I really think.
	Difficult to answer questions or concerns about ACP when asked openly.
Realize as much with familys' connection.	Feeling the connection strengthened by listening to family members' true thoughts and feelings.
	Concerned that communicating my worries is a burden to my family

Additionally, patients were satisfied that they had made the best choices they could make through the process. In reflecting on this experience after surgery, patients spent a lot of time thinking about their families and reflecting on their wishes for treatment and their future lives. Patients were satisfied that they had the opportunity to express their wishes to their family members and felt connected to them. The relationships between patients and their families were deemed complex. The patients were concerned that communicating their concerns to family members would make them feel burdened. On the other hand, patients recognized that organizing and sharing their wishes for life-sustaining treatment was important for their families and tried to create opportunities for them to discuss their wishes with their families. Furthermore, they recognized the need to consider ACP not only before surgery, but also thereafter.

After discharge, they expressed the need to make joint decisions with medical professionals regarding future treatment. The experience of considering ACP preoperatively has led to an awareness of the need to choose the type of care patients wants to receive based on their values. They also indicated the need for ACP support as part of routine perioperative care. Even during the perioperative period when recovery is a prerequisite, it is important for patients to feel that they can discuss life-sustaining treatments.

Families’ experiences

Family experiences are demonstrated in Table 3. Eight categories and 34 subcategories were extracted in two phases: before surgery and after discharge. In the prehospital phase, all families tended to avoid the topic even though they wanted to ask patients about their wishes or thoughts about surgery and ACP. Families then tried to follow the pace of the patients who dared to behave well. Moreover, they worried that by talking about ACP and surgery, patients would lose hope of living. Furthermore, families perceived the necessity for patients to undergo surgery as an overwhelmingly painful reality, leading them to cope with the distress independently.

**Table 3:** Families’ experiences regarding advance care planning for patients before surgery ACP, advance care planning; DA, decision aid.

Category	Subcategory
Before surgery	
I care about my patients, but I can't say a word, so I give a semblance of usual life	Check-in with the patient to see how he or she is feeling.
	Adjust to the pace of the patient's cheerful demeanor.
	Find a scene to start a conversation.
I keep my unspoken thoughts to myself only	I have difficulty expressing my opinion in front of my patients.
	I just accept what the patient decides.
	I am afraid that my words will hurt or be misunderstood by the patient.
	I hope patients do not lose hope.
Look for what I can do to help the patient	Strive to accept the reality that is too painful for me.
	Avoid topics I don't want to think about.
	All I can do is believe.
	Realize how much the patient means to me.
Share information about the patient among family members or share my role	I discuss the patient's post-treatment imaginings while keeping my emotions in check.
	Find out the differences in thinking among family members due to their different positions (ex: partner, child).
	In discussions among family members, discuss the worst-case scenario for the patient.
	Reorganize roles among family members in case the patient dies.
	Think about who would bear this responsibility and keep their mouths shut.
After discharge	
Understanding my awareness and responsibility as a surrogate decision-maker	Understand my role when I am a surrogate decision-maker.
	I am not confident that I would be able to communicate the patient's wishes to the physician or make decisions on behalf of the patient in an actual crisis situation.
	I am not confident that I can communicate the decision to "withdraw/withhold from treatment" in a surrogate position.
	Feel guilty about withdrawing/withholding treatment even if it is the patient's intention.
	Cannot understand the reasons for the thoughts' reasons from the patient's written intentions.
Seeking support for me from medical providers	Want to start discussing ACP with patients, including with medical providers.
	Want support for family during patient's hospitalization.
	Difficult to express true feelings in discussions between patient and family only.
	Want medical provider to accompany the patient in the ACP decision-making process.
Relieved at the success of a patient's treatment and thinking about the next treatment	Caring for patients through video calls during hospitalization.
	Relief that the worst was averted.
	Worried about post-operative treatment and recurrence.
Learn about ACP and make plans for me and patient's life	Understand the ACP process myself as I use the DA with patient.
	The topic of ACP becomes an important topic for me and our family.
	Understand that the topic of ACP is an important topic for me as well.
	Realize that a change in position will change how and what decisions are made.
	Become more willing to prepare for situations where life-sustaining treatment is necessary.
	Use DA to guide the ACP's decision-making direction.
	Realize my values as a family member.

## Discussion

The study visualized patients’ and families’ experiences upon initiating ACP support before surgery. This may help in considering methods of ACP support for patients who are not at the end of their lives and in identifying evaluation indicators. Patients learned about ACP based on this experience. They also understood the meaning of ACP and began to continuously think about the treatment they wanted to receive based on their wishes after the treatment. In addition, patients reflected that discussing their values and treatment preferences with their families allowed them to make better decisions regarding ACP after treatment. Providing ACP support at this time might help patients make decisions about medical care that meet their needs, including the desire for resuscitation care in the event of an emergency [7,11]. Before receiving high-risk treatment, patients, their families, and medical providers could feel comfortable talking about life-sustaining treatment in anticipation of the worst risks, thereby strengthening informed consent [19,20]. When patients understand the purpose of ACP and begin discussions with their medical providers and families, they may be able to share their true feelings and provide patient-centered care. Repeating and brushing up on ACP discussions at the patient's pace may also lead to more satisfactory support for patients and families. Furthermore, when patients and their families express their thoughts, and the medical provider comprehends the content, it becomes feasible to make medical choices aligning with the patient's values, thereby promoting shared decision-making (SDM) [16,21]. The process of continuing attendance often persists after patients' complete surgery and are discharged [22]. It is also necessary for medical providers to be creative in including ACP support as part of their regular medical care delivery while observing changes in patients' and family members' feelings during the treatment process. On the other hand, it has become obvious that it is difficult for patients and their families to openly discuss their wishes while assuming end-of-life care. Thus, patient-family discussions may require assistance from medical counseling and coaching. Before treatment, patients and their families often found it challenging to discuss their anxieties, and each party dealt with these concerns independently. It is important to support patients and their families to express their concerns prior to treatment. ACP is a complex process that occurs over time and consists of various behaviors and interactions [23]. There are limitations to determining the effectiveness of ACP support using only a single method or evaluation [23,24]. The outcomes of ACP support for perioperative patients and patients treated in emergency and ICU settings include increased knowledge [25] and perceptions of quality of life [26]. Based on the results of this study, it may be necessary not only to create opportunities for patients and providers to discuss ACP but also to create opportunities for patients and families to engage in dialogue regarding ACP and to assess its content, quality, and satisfaction.

In Japan, the average length of stay in acute-care hospitals is 11.8 days [27]. At the study institution, the length of hospital stays for patients who underwent high-risk surgery was comparable and, in fact, shorter. In acute care hospitals, patients undergoing high-risk surgery and their families have the opportunity to engage with several medical providers in various settings, including outpatients, general wards, operating rooms, and ICUs [9]. The study also found that, for patients who engage with many providers in a short time, finding a provider they trust and taking the time to talk about their values and treatment intentions could pose a limitation. Providing ACP support is limited only to physicians who perform informed consent. To address this, the utilization of nurses, including advanced practice nurses, is explored [28]. It may prove effective to involve patients and their families in ACP discussions from the early stages of treatment [23]. Based on the physicians’ IC, it is important to support the patients and their families when making decisions based on their understanding of explanations. In decision-making regarding ACP, patients are affected by various factors, including their relationships with their family members and medical providers. Medical providers should help patients autonomously, based on their understanding of these issues. Medical providers should also adopt a communicative approach and coordinate opportunities for discussion at the right time with patients and their families.

Limitations

This study included only one institution and is limited by the generalizability of the study data. In addition, during the study period, family visits were restricted for the patient's hospitalization to control COVID-19, which was not part of the usual medical system. This may have limited opportunities for face-to-face discussions between patients and their families. Additionally, the intervention and interviews were conducted by a single researcher, so interviewer bias may exist. However, the evaluation and analysis of the interview data were conducted by multiple supervisors to minimize bias.

## Conclusions

ACP support before high-risk surgery may be an introductory phase for patients and their families to familiarize themselves with its concept. In addition, the associated risks of treatment could prompt patients to contemplate the type of treatment they desire and how after the quality of life they envision post-treatment. This may encourage patients to think independently about their treatment and discuss it with their physicians in order to promote SDM.

However, it became clear that it was difficult for the patients and their families to openly discuss their wishes and thoughts regarding end-of-life treatment. For better assistance, ACP support should be incorporated in addition to usual care even before a new treatment is initiated. In the event of an emergency, this can lead to surrogate decision-making that respects the patient's autonomy. An important outcome of ACP support for patients undergoing surgery and intensive care may be enhanced SDM, increased discussion between the patient and family, and concordance of intentions. In addition, a reduction in the mental burden on medical providers may be evaluated as a secondary effect.
